# The Role of iRhom2 in Metabolic and Cardiovascular-Related Disorders

**DOI:** 10.3389/fcvm.2020.612808

**Published:** 2020-11-24

**Authors:** Ramasatyaveni Geesala, Priya D. Issuree, Thorsten Maretzky

**Affiliations:** ^1^Inflammation Program, Department of Internal Medicine, Roy J. and Lucille A. Carver College of Medicine, University of Iowa, Iowa City, IA, United States; ^2^Department of Internal Medicine, Holden Comprehensive Cancer Center, Roy J. and Lucille A. Carver College of Medicine, University of Iowa, Iowa City, IA, United States

**Keywords:** cardiovascular disease, obesity, diabetes, tumor necrosis factor (TNF), epidermal growth factor receptor (EGFR), a disintegrin and metalloproteinase 17 (ADAM17), rhomboid 5 homolog 2 (RHBDF2), inactive rhomboid 2 (iRHOM2)

## Abstract

Chronic obesity is associated with metabolic imbalance leading to diabetes, dyslipidemia, and cardiovascular diseases (CVDs), in which inflammation is caused by exposure to inflammatory stimuli, such as accumulating sphingolipid ceramides or intracellular stress. This inflammatory response is likely to be prolonged by the effects of dietary and blood cholesterol, thereby leading to chronic low-grade inflammation and endothelial dysfunction. Elevated levels of pro-inflammatory cytokines such as tumor necrosis factor (TNF) are predictive of CVDs and have been widely studied for potential therapeutic strategies. The release of TNF is controlled by a disintegrin and metalloprotease (ADAM) 17 and both are positively associated with CVDs. ADAM17 also cleaves most of the ligands of the epidermal growth factor receptor (EGFR) which have been associated with hypertension, atherogenesis, vascular dysfunction, and cardiac remodeling. The inactive rhomboid protein 2 (iRhom2) regulates the ADAM17-dependent shedding of TNF in immune cells. In addition, iRhom2 also regulates the ADAM17-mediated cleavage of EGFR ligands such as amphiregulin and heparin-binding EGF-like growth factor. Targeting iRhom2 has recently become a possible alternative therapeutic strategy in chronic inflammatory diseases such as lupus nephritis and rheumatoid arthritis. However, what role this intriguing interacting partner of ADAM17 plays in the vasculature and how it functions in the pathologies of obesity and associated CVDs, are exciting questions that are only beginning to be elucidated. In this review, we discuss the role of iRhom2 in cardiovascular-related pathologies such as atherogenesis and obesity by providing an evaluation of known iRhom2-dependent cellular and inflammatory pathways.

## Introduction

Cardiovascular diseases (CVDs) remain the leading causes of deaths worldwide; nearly 18 million people die from CVDs each year (1, 2). CVDs describe a range of conditions that affect the structure and function of the heart, consisting of various types such as coronary artery disease, congenital heart disorder, heart failure, and vascular diseases (3). In addition to hypertension and other CVD risk factors such as hypercholesterolemia, obesity has consistently been associated with an increased risk for CVD-related morbidity and mortality (4, 5). Obesity is a consequence of genetic and environmental factors that promote an imbalance in energy consumption and expenditure, leading to excessive fat accumulation in the adipose tissue, the body's primary repository for energy (6, 7). This fat accumulation can directly contribute to CVDs through reduced insulin sensitivity, impaired insulin production, decreased glucose uptake in various tissues, and obesity-associated low-grade inflammation (8, 9).

Chronic low-grade inflammation of adipose tissue is characterized by activation of adipose tissue macrophages and infiltration of circulating macrophages that surround enlarged and dying adipocytes to form crown-like structures, a characteristic indicator of adipose inflammation (10), as well as infiltration of other immune cells (11, 12), leading to increased production of cytokines such as tumor necrosis factor (TNF) (13). These increased levels of adipose tissue-derived TNF have been suggested to alter lipid storage, oxidative capacity and adipose tissue expansion (14). In addition to its role in modifying adipose tissue function and expandability, enhanced production of TNF can also induce vascular insulin resistance, which in turn promotes premature endothelial cell death and premature aging of coronary arteries (15).

TNF is released upon proteolytic shedding by a disintegrin and metalloprotease (ADAM) 17 (16–19). In addition to TNF, ADAM17 also cleaves several ligands of the epidermal growth factor receptor (EGFR) (20, 21). While ligand-dependent EGFR signaling is critical for mammalian cardiogenesis (22), its function in the vasculature and its role in CVDs are less well-understood (23). In blood vessels, endothelial cells and vascular smooth muscle cells are both producers and cellular targets of EGFR ligands such as amphiregulin (AREG) and heparin-binding EGF-like growth factor (HB-EGF) (24). EGFR signaling has also been associated with arterial blood pressure regulation, vascular remodeling, and arteriosclerosis (24). Furthermore, increased levels of circulating EGFR ligands may promote cardiac hypertrophy and diabetic cardiomyopathy associated with chronic low-grade inflammation (25–27). Meanwhile, the role of EGFR signaling in obesity and obesity-induced metabolic syndrome is currently unclear.

Furthermore, ADAM17 mediates the release of angiotensin converting enzyme 2 (ACE2) from cardiomyocyte membranes, vascular smooth muscle cells, and endothelial cells, resulting in increased plasma ACE2 activity, a known marker of adverse prognosis in patients with CVD (28). In addition, ADAM17 can cleave various cell surface molecules including interleukin (IL) 6 receptor, vascular cell adhesion molecule 1, and L-selectin, which facilitates infiltration of leukocytes through the vasculature into adipose tissue (29–31). The rhomboid 5 homolog proteins iRhom1 and iRhom2 (also known as RHBDF1 and RHBDF2) are unique members of the rhomboid family and act as important modulators of ADAM17 activity (32, 33). They are not only involved in the translocation of ADAM17 to the outer membrane but also regulate its maturation, activation and substrate selectivity (34). Interestingly, iRhom2 deficiency results in the loss of ADAM17-mediated shedding events in hematopoiesis-related tissues, such as bone marrow and lymph nodes, while the close homolog, iRhom1, supports the critical functions of ADAM17 in most other non-immune cells and tissues (17, 19, 35).

By being a crucial and tissue specific regulator of ADAM17, iRhom2 has emerged as a novel target molecule for selective inactivation of ADAM17. However, the role of iRhom2 in the pathogenesis of CVDs is only beginning to be explored. In this review, we summarize the findings and plausible mechanisms that underlie the role of iRhom2 in the pathogenesis of obesity-related CVDs. Of note to the readership, the trafficking and maturation of ADAM10, another ubiquitously expressed transmembrane metalloprotease that principally modulates Notch signaling in various cell types is not dependent on iRhoms but on a subgroup of tetraspanins (36, 37). For a thorough and comprehensive molecular understanding of these transmembrane 4 superfamily proteins and their interacting partners, we refer the interested readers to reviews by Charrin and Matthews (38, 39).

## From Rhomboids to ADAMs: The Essential Regulators of EGFR Signaling

Rhomboid proteases belong to a group of intramembrane serine proteases that mediate the proteolytic processing of membrane anchored EGFR-ligands in flies (40). They consist of six transmembrane-spanning helices and a catalytic site that depends on a serine and histidine catalytic dyad (41). In mammals, most EGFR ligands are proteolytically cleaved by the membrane-anchored disintegrin and metalloprotease ADAM17, which is structurally very distinct from rhomboids (42). ADAM17 belongs to a family of type-I transmembrane proteins with an ectodomain comprising of a pro-peptide, a catalytic- and disintegrin domain as well as a membrane-proximal region followed by a transmembrane helix and a cytoplasmic tail (36, 43). A structure-function link between the rhomboid family and ADAM17 was first suggested when the proteolytically inactive rhomboid protein, iRhom2, was identified as a key regulator of the ADAM17 maturation and trafficking to the cell surface in immune cells (32, 33). Further insight into the relationship between ADAM17 and iRhom2 was provided by a missense mutation in the first transmembrane segment of iRhom2, called sinecure, which results in reduced cleavage of TNF from bone marrow-derived macrophages (BMDMs) (44). Mice that are homozygous for the iRhom2 sinecure mutation and also deficient in the related iRhom1 display a similar phenotype as mice that are deficient for iRhom1 and iRhom2 (45), highlighting the significant functional impact of the sinecure point mutation associated with ADAM17 function during development.

In addition to its role in ADAM17 maturation in different cell types of the immune system, iRhom2 also governs the substrate selectivity of a wide range of structurally different ADAM17 substrates, including the ephrin receptor EPHB4, the pleiotropic cytokine KITL2, the angiopoietin receptor TIE2, and several of the EGFR-ligands (34). For instance, while the EGFR ligand, transforming growth factor alpha (TGFα), is shed by ADAM17 in an iRhom2-independent manner, ADAM17-mediated shedding of EGFR ligands, such as HB-EGF and AREG, is critically dependent on iRhom2 (34). Although it is currently not known how iRhom2 confers substrate selectivity for ADAM17-mediated shedding events, it has been shown that the extracellular juxtamembrane domain (JMD) of iRhom2 is required for the rapid activation of ADAM17 (46, 47). Further studies found that the main determinants for PMA-stimulated shedding of iRhom2-dependent substrates such as AREG reside in its JMD and transmembrane domains (TMD) and not in the EGF-like modules, suggesting that the substrate's JMD and TMD are important for its selective stimulated shedding by iRhom2/ADAM17 (34). However, it remains unclear how iRhom2 interacts with its structurally diverse clients in a regulated manner, ensuring substrate selectivity to stimulated shedding by ADAM17. Moreover, individual point mutations in the transmembrane domain of ADAM17 that were predicted to disrupt the interactions with the first transmembrane segment of iRhom2 selectively reduce iRhom2/ADAM17-dependent ectodomain shedding, without affecting the iRhom1/ADAM17-dependent proteolysis of cell surface molecules (45). Taken together, the different substrate preferences of iRhom1 and iRhom2 in ADAM17-mediated ectodomain shedding (34), the impact of the sinecure mutation on ADAM17 (44), and the consequences of point mutations in the transmembrane domain of ADAM17 on iRhom2-dependent shedding events (45) support a model whereby iRhom2 and ADAM17 function as a heteromeric complex (48). The formation of this complex is likely initiated in the endoplasmic reticulum and remains stable throughout trafficking to regulate the proteolytic cleavage of iRhom2/ADAM17-dependent substrates on the cell surface or in the late secretory pathway. This model is further supported by the finding that mutations in the phosphorylation sites of its cytoplasmatic domain affect the activity of ADAM17 (49, 50).

iRhom2 has also been reported to interact with several cytoplasmic molecules, including the stimulator of interferon genes (STING) (51). While initial studies implicated iRhom2 in the regulation of STING protein stability and activation-induced translocation from the endoplasmic reticulum to perinuclear microsomes, it has now been demonstrated that endogenous iRhom2 is not required for STING stability but is essential for ADAM17 stability (48, 52).

These observations support the hypothesis that ADAM17 is a principal interaction partner of iRhom2 and that this partnership ensures the successful maturation and function of ADAM17 on the cell surface (48). However, additional molecular players that promote or impede the stability of iRhom2 and ADAM17 under homeostatic and inflammatory conditions remain to be uncovered. Recently, Frmd8 (FERM domain containing 8), also termed iTAP (iRhom tail associated protein), an iRhom2-interacting partner, was reported to regulate the lysosomal degradation of iRhom2 and ADAM17 (53, 54). Intriguingly, Frmd8 is highly expressed in various populations of immune cells^1^ and future work will determine whether its expression levels can modulate iRhom2/ADAM17 activity and impact inflammatory signaling pathways in disease models. A list of discussed and currently known iRhom2 client proteins and its interacting partners is summarized in Table 1.

**Table 1:** iRhom2 binding partners and clients.

**iRhom2 interaction partners/targets**	**Abbreviation**	**References**
14-3-3 protein	14-3-3	(49, 50)
A disintegrin and metalloprotease 17	ADAM17	(32, 33, 44)
Amphiregulin	AREG	(34)
Angiopoietin-1 receptor	TIE2, CD202B	(34)
Colony stimulating factor 1	CSF1, M-CSF	(55)
Colony stimulating factor 1 receptor	CSFR1, M-CSFR	(56)
EPH receptor B4	EPHB4	(34)
Epiregulin	EREG	(34)
Heparin-binding EGF-like growth factor	HB-EGF	(34)
iRhom tail-associated protein	iTAP/FRMD8	(53, 54)
KIT ligand 2	KITL2	(34)
Nuclear factor E2-related factor 2	NRF2	(57)
Transformation-related protein 63	p63	(58)
Peroxisome proliferator-activated receptor γ	PPARγ	(59)
Stimulator of interferon genes	STING	(51)
Tumor necrosis factor	TNF	(32, 33, 44)
Tumor necrosis factor receptors	TNFRs	(33, 60)
Human cytomegalovirus tegument protein UL82	UL82	(61)
Virus-induced signaling adaptor	VISA	(62)

## The Role of iRhom2 in Obesity-Induced Low-Grade Inflammation and Metabolic Syndrome

Obesity is a chronic and often progressive disease, similar to hypertension or type 2 diabetes (63). In addition to genetic predisposition that can promote accumulation of fat, a variety of environmental factors related to nutrient composition, the gut microbiome, and sedentary habits have been implicated in the pathogenesis of obesity (64–66). Mechanistically, obesity is a consequence of a sustained positive energy balance between energy intake and energy expenditure. This leads to a pathological increase in fat mass through hypertrophy (cell size enlargement) and hypoplasia (cell number decrease) of adipocytes as well as increased fat deposition in the myocardium (67–69). Abnormal fat accumulation can promote peripheral insulin resistance through various mechanisms, including the dysregulation of lipid molecules such as ceramides (70) and branched-chain amino acids (71), which together culminate in an increased probability of type 2 diabetes and metabolic syndrome development (8). In addition, low-grade inflammation in adipose tissue has been associated with several obesity-associated complications including insulin resistance and type 2 diabetes (72).

**Figure 1:**
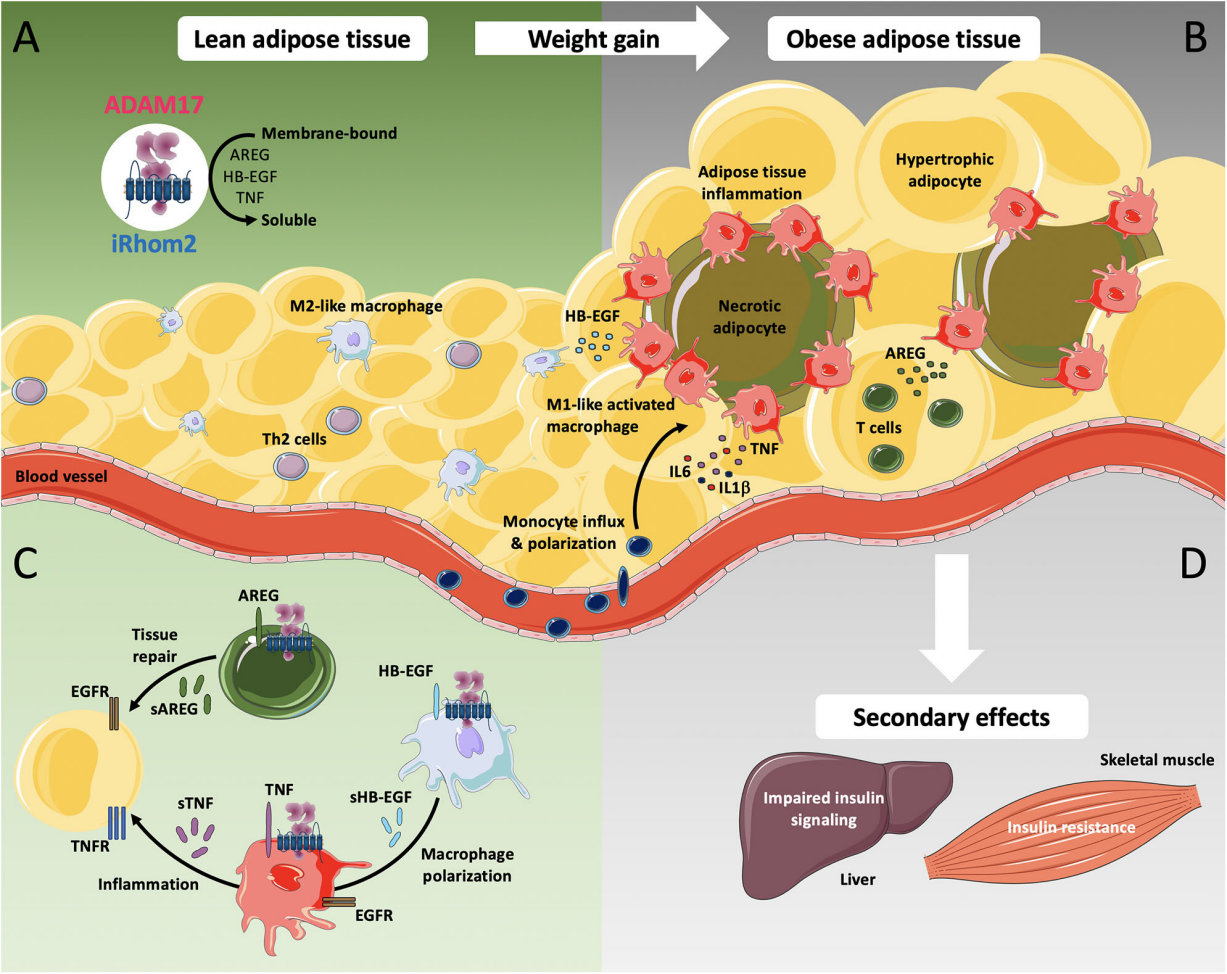
iRhom2-dependent regulation of ADAM17 in lean adipose tissue and metabolic activation during chronic adiposity. ADAM17 is widely expressed in several cell types including adipocytes, adipose tissue fibroblasts, smooth muscle cells, and adipose tissue-associated immune cells. **(A)** ADAM17-mediated shedding of the pro-inflammatory cytokine TNF as well as the release of EGFR-ligands such as amphiregulin (AREG) and heparin-binding EGF-like growth factor (HB-EGF) requires the presence of its cofactor iRhom2. In lean adipose tissue, Th2 cells as well as other cell types produce type 2 cytokines, including IL4, IL5, and IL13 and play an important role in M2-like macrophage polarization. M2-like macrophages facilitate rapid removal of apoptotic adipocytes and regulate adipocyte lipolysis by taking up and storing excessive amounts of adipocyte-released lipids, directing gradual lipid release into blood circulation. **(B)** As adiposity increases, obese adipose tissue displays adipocyte hypertrophy, which leads to hypoxia, intracellular stress and lipotoxicity, resulting in excessive secretion of pro-inflammatory cytokines including TNF, IL6, and IL1β. Increased monocyte influx and M1-like macrophage polarization undergo a drastic change in distribution, resulting in the formation of crown*-*like structures around necrotic adipocytes. **(C)** Additionally, increased production of AREG in obese adipose tissues possibly induces compensatory mechanisms against aberrant accumulation of lipids while production of HB-EGF potentially promotes polarization of M1-like macrophages to M2-like macrophages. **(D)** The secondary effects of obesity can lead to impaired insulin signaling, progressive hepatic dysfunction in the liver as well as to accumulation of intramyocellular lipids, leading to insulin resistance in the skeletal muscle. Adapted cell and tissue images are taken from Servier Medical Art, under Creative Commons Attribution 3.0 Unported License; https://smart.servier.com.

Previous studies have indicated that increased recruitment of macrophages in adipose tissue and their polarization into a pro-inflammatory M1-like phenotype is a key contributor to development of obesity-associated low-grade inflammation (Figure 1). These macrophage populations express high levels of pro-inflammatory cytokines such as IL1β and TNF, and previous studies have shown that deficiency in TNF as well as TNF neutralization can improve glucose and insulin tolerance in mouse models of obesity (73–75). However, clinical studies have shown that blockade of TNF signaling alone is insufficient for improving insulin resistance or endothelial function in obese humans, suggesting that other pathways are involved in obesity-associated metabolic dysregulation (76).

Our laboratory has shown that mice lacking iRhom2 are protected from high-fat diet (HFD)-induced adipose tissue inflammation and show significantly less macrophage infiltration as well as reduced levels of pro-inflammatory cytokines in adipose tissue (77). However, *iRhom2*-deficient mice gain substantially more weight than wild type mice on calorie-rich diet, suggesting that the lack of iRhom2 contributes to the expansion of adipose tissue mass in obesity.

Concordant with the role of iRhom2 in regulating inflammatory processes and adipose tissue homeostasis during obesity (77), activation of ADAM17 has been shown to promote metabolic inflammation, presumably through the release of inflammatory cytokines such as TNF and regulation of pathways involved in adipose tissue infiltration by immune cells (78, 79). In addition, *in vitro* studies have implied a role for ADAM17 in adipocyte differentiation and lipogenesis (79, 80), but its physiological role in this context is still unknown. These findings support the idea that the observed detrimental effects of obesity in *iRhom2*-deficient mice could be at least partly due to a failure of iRhom2-dependent activation of ADAM17-mediated signaling events.

In adipose tissue, AREG expression is markedly upregulated during HFD-induced obesity in wild type mice but is profoundly downregulated in *iRhom2*-deficient mice, suggesting a possibly leading role of EGFR-dependent cellular processes over TNF in the modulation of metabolic pathways during obesity (77). Alternatively, it is possible that crosstalk between TNF and EGFR signaling in particular cell types is critical for the modulation of metabolic pathways, as seen in other disease models (52). Although the role of AREG during obesity development is still unclear, its production in adipose tissues could presumably act as a compensatory regulator against aberrant accumulation of white adipose tissue and downregulate adipose tissue mass through enhanced energy expenditure or lipolysis (81). While chronic inflammation has been linked to obesity and its pathophysiological consequences, these findings suggest that pro-inflammatory cytokine-mediated signaling alone or in combination with EGFR-dependent mechanisms in adipose tissue are potentially required for proper remodeling and expansion. Consistent with our findings and interpretation, and in contrast to the common dogma that the link between inflammation and metabolic syndrome is a linear one, recently and prior published studies have shown that suppressing adipocyte inflammation can hinder normal adipose tissue function and drives insulin resistance, despite having some benefits on weight gain (82, 83).

Interestingly, recent studies using an independently generated line of *iRhom2*-deficient mice, came to different conclusions regarding the role of iRhom2 in diet-induced obesity and metabolic complications of HFD (84, 85). In particular, Badenes et al. (84) have reported that loss of iRhom2 protects mice from weight gain, chronic inflammation in adipose tissues, hepatic steatosis and insulin resistance when challenged by a high-calorie diet. Moreover, Xu et al. (85) speculated that loss of iRhom2 from macrophages in adipose tissues could reduce inflammation and improve insulin sensitivity by suppressing paracrine interactions between macrophages and fat cells. Although it is currently unknown what molecular mechanisms are responsible for the differences between these studies, Badenes et al. (84) observed increased thermogenesis via brown adipose tissue activation and beige adipocyte recruitment in the absence of iRhom2, allowing *iRhom2*-deficient mice to disperse excess energy more efficiently than wild type mice. While these effects on body weight responses to HFD are in conflict with the phenotype reported by Skurski et al. (77), a common feature could be the enhanced thermogenesis in obese *iRhom2*-deficient mice, which was counter-balanced by the reduced movement vigor in the study by Skurski et al. (77). Although the published details of how the *iRhom2*-deficient mice used by Xu et al. (85) were generated are unclear (85, 86), there are differences between the approaches utilized by Badens et al. (84) and Skurski et al. (77) to generate *iRhom2*-deficient mice, namely in the targeting constructs and strain of embryonic stem cells used, as described in the *Methods* section in the respective publications (32, 33). While both studies initially reported impaired TNF signaling as a result of iRhom2 deficiency, it is plausible the aforementioned differences act as variables that potentially affect the outcome seen in these mice. Additional studies will be necessary to identify possible modifier genes and/or environmental factors, including microbiota and housing differences, that could further explain the causes of such phenotypic discordance.

## iRhom2 Function is Crucial for Myocardial Regeneration and Vascular Homeostasis

TNF is a key and well-studied pro-inflammatory cytokine tagged as a potential biomarker and therapeutic target in CVDs (87–90). Indeed, previous studies have reported a strong relationship between circulating soluble TNF and mortality in patients with heart failure (91). However, anti-TNF treatment with the soluble TNF receptor (TNFR) etanercept, or with infliximab, a neutralizing TNF antibody, have been unsuccessful in clinical trials and have shown a worsening of heart failure in some cases (88, 89, 92). These studies have hinted at a potential role for the pro-inflammatory response as being a reacting tissue repair mechanism that is considered to provide beneficial functions by supporting cardiac remodeling early during cardiac stress. Indeed, during normal cardiac function, TNF-mediated signaling exerts important effects on the cardiovascular system to regulate vascular permeability and glucose homeostasis as well as extracellular matrix formation and cardiomyocyte apoptosis (93). However, the paradoxical role of TNF as being both cardioprotective and deleterious remains overall contentious, partly due to the different animal models used to drive injury (94–97). Furthermore, due to its pleiotropic actions and differential signaling mediated by two functionally distinct receptors, TNFRs 1 and 2, the role of TNF in this process has been challenging to ascertain. Notably, both ligand and receptors are initially expressed as transmembrane proteins regulated through iRhoms/ADAM17-mediated proteolytic processing.

Due to the reliance of immune cells on iRhom2 for TNF production (19, 33), *iRhom2*-deficient mice have been used as a genetic model to assess the contribution of immune-cell derived TNF during cardiac injury. In a surgical ligation mouse model of myocardial infarction, it was recently shown that iRhom2-mediated TNF signaling is involved in tissue repair and wound healing in the myocardium following myocardial infarction (98). iRhom2 was shown to regulate TNF signaling and pro-inflammatory pathways in classically activated M1-like macrophages, which promote tissue regeneration and collagenous scar formation following myocardial infarction (98). The observation that iRhom2 and immune cell-derived TNF are required for reparative connective tissue formation in response to injury during myocardial infarction implies that the healing process is not exclusively mediated by M2-like macrophages and that macrophages with pro-inflammatory signatures may play a significant role in mediating cardiac repair. These observations emphasize the potential crosstalk between the pro-inflammatory and repair stages following myocardial infarction. In contrast, Lu et al. (99) have shown that iRhom2-mediated inflammation in an LPS-induced heart injury model can drive cardiac injury via regulation of the Toll-like receptor signaling pathway. This finding is perhaps unsurprising, given the reliance of the LPS-induced heart injury model on soluble TNF to induce injury (99–101), thus making interpretation of the role of TNF on infarct repair difficult in this model. The deleterious and beneficial roles of iRhom2-dependent TNF signaling could be dose- and context-dependent and in part associated with specific receptor subtype-mediated effects. Indeed, earlier studies elucidating the pro-inflammatory response after myocardial infarction have mainly focused on TNF signaling and its role in late-stage remodeling, infarct size and cardiac output (95–97), and have only superficially investigated the role of TNF in regulating cardiac immune cell phenotypes or functions. Meanwhile, the specific role of iRhom2 in atherosclerosis is still unclear and an area of active investigation. Increased iRhom2 expression has been positively correlated with elevated macrophage-associated inflammation and accumulation of oxygen reactive species in atherosclerotic lesions induced by high-calorie diet and has been proposed to promote atherosclerosis through macrophage activation and the induction of oxidative stress (102, 103). Although this remains to be tested genetically, in an *apolipoprotein E*-deficient mouse model of atherosclerosis, it has been shown that myeloid-specific deletion of ADAM17 resulted in significant increases in atherosclerosis development with a decrease in lesional macrophage area while endothelial-specific ADAM17 deficiency led to reduced atherosclerosis development (104). Therefore, examining the role of iRhom2 in this disease will shed light on the potential role of ADAM17 and its unique substrates.

## Summary: The Goldilocks Principle of iRhom2 Expression

**Figure 2:**
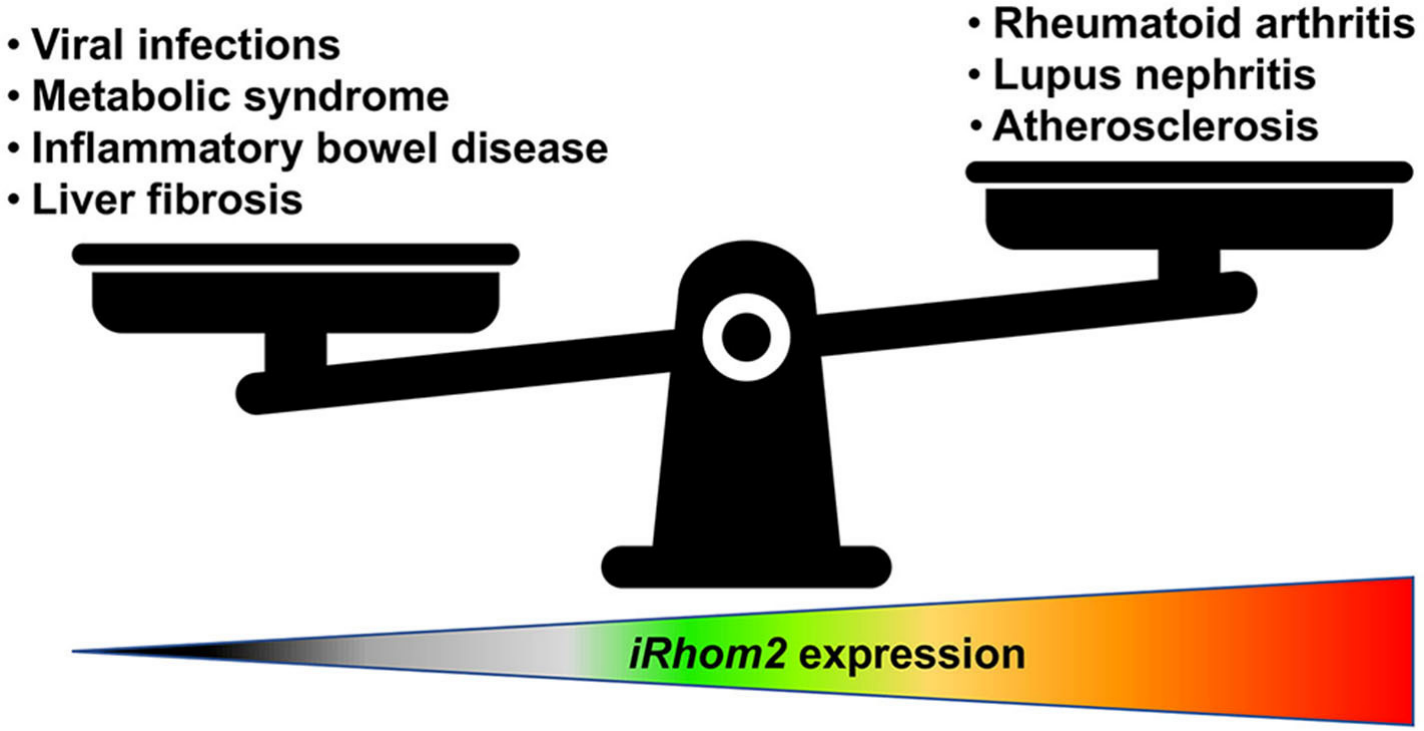
iRhom2 function is a matter of proportion. iRhom2 regulates various shedding events of numerous cell surface transmembrane proteins such as release of the pro-inflammatory cytokine TNF and processing of EGFR ligands, including amphiregulin and heparin-binding EGF-like growth factor. Under normal conditions, this proteolytic cleavage is necessary for cell movement and tissue remodeling in the intestine and adipose tissue. *iRhom2*-deficient mice are viable and display no developmental defects unless they encounter viral or bacterial pathogen challenges that rely on effective TNF signaling. In addition, iRhom2 exhibits a critical role in obesity-related metabolic disorders for its involvement in the regulation of inflammation, glucose uptake, and insulin resistance. However, increased expression or activity of iRhom2 can contribute to atherosclerotic cardiovascular diseases as well as autoimmune diseases such as lupus nephritis or rheumatoid arthritis.

Although excessive and sustained expression of iRhom2 has been linked to worse outcomes in various inflammatory disease models [for detailed reviews on iRhom2 in diseases, we direct the reader to (42, 105)], this catalytically inactive member of the rhomboid family of intramembrane serine proteases may have very important homeostatic roles as a potential regulator of protective and beneficial low-level inflammatory responses in the host (77, 98, 106) (Figure 2). Therefore, uncovering novel modulators of iRhom2 expression and stability during homeostasis and disease will allow for considerable advancements in therapeutic interventions. Furthermore, given its intriguing role in coupling substrate selectivity with modulation of ADAM17 activity, further studies dedicated to elucidating tissue-specific ligand availability in disease and homeostasis will be instrumental. Undoubtedly, we have much more to learn about the biology of iRhom2 and its complex interaction with its clients before we can truly appreciate its role in health and disease.

## Author Contributions

RG, PI, and TM: writing—review and editing. All authors contributed to the article and approved the submitted version.

## Conflict of Interest

The authors declare that the research was conducted in the absence of any commercial or financial relationships that could be construed as a potential conflict of interest.

## Footnotes

^1^http://biogps.org/#goto=genereport&id=67457
